# The Distribution and Associated Factors of HIV/AIDS Among Youths in Guangxi, China, From 2014 to 2021: Bayesian Spatiotemporal Analysis

**DOI:** 10.2196/53361

**Published:** 2024-09-27

**Authors:** Juntong Li, Runxi Zhang, Guanghua Lan, Mei Lin, Shengkui Tan, Qiuying Zhu, Huanhuan Chen, Jinghua Huang, Dongni Ding, Chunying Li, Yuhua Ruan, Na Wang

**Affiliations:** 1 Guangxi Key Laboratory of Environmental Exposomics and Entire Lifecycle Health, School of Public Health, Guilin Medical University, 1 Zhiyuan Road, Guilin, 541100, China, 86 07733680605; 2 Guangxi Key Laboratory of Major Infectious Disease Prevention Control and Biosafety Emergency Response, Guangxi Center for Disease Control and Prevention, 18 Jinzhou Road, Nanning, China; 3 State Key Laboratory of Infectious Disease Prevention and Control (SKLID), Chinese Center for Disease Control and Prevention (China CDC), Collaborative Innovation Center for Diagnosis and Treatment of Infectious Diseases, Beijing, China

**Keywords:** HIV/AIDS, acquired immunodeficiency syndrome, youth, reported incidence, Bayesian model, spatiotemporal distribution

## Abstract

**Background:**

In recent years, the number of HIV/AIDS cases among youth has increased year by year around the world. A spatial and temporal analysis of these AIDS cases is necessary for the development of youth AIDS prevention and control policies.

**Objective:**

This study aimed to analyze the spatial and temporal distribution and associated factors of HIV/AIDS among youth in Guangxi as an example.

**Methods:**

The reported HIV/AIDS cases of youths aged 15‐24 years in Guangxi from January 2014 to December 2021 were extracted from the Chinese Comprehensive Response Information Management System of HIV/AIDS. Data on population, economy, and health resources were obtained from the Guangxi Statistical Yearbook. The ArcGIS (version 10.8; ESRI Inc) software was used to describe the spatial distribution of AIDS incidence among youths in Guangxi. A Bayesian spatiotemporal model was used to analyze the distribution and associated factors of HIV/AIDS, such as gross domestic product per capita, population density, number of health technicians, and road mileage per unit area.

**Results:**

From 2014 to 2021, a total of 4638 cases of HIV/AIDS infection among youths were reported in Guangxi. The reported incidence of HIV/AIDS cases among youths in Guangxi increased from 9.13/100,000 in 2014 to 11.15/100,000 in 2019 and then plummeted to a low of 8.37/100,000 in 2020, followed by a small increase to 9.66/100,000 in 2021. The districts (counties) with relatively high HIV/AIDS prevalence among youths were Xixiangtang, Xingning, Qingxiu, Chengzhong, and Diecai. The reported incidence of HIV/AIDS among youths was negatively significantly associated with road mileage per unit area (km) at a posterior mean of −0.510 (95% CI −0.818 to 0.209). It was positively associated with population density (100 persons) at a posterior mean of 0.025 (95% CI 0.012‐0.038), with the number of health technicians (100 persons) having a posterior mean of 0.007 (95% CI 0.004‐0.009).

**Conclusions:**

In Guangxi, current HIV and AIDS prevention and control among young people should focus on areas with a high risk of disease. It is suggested to strengthen the allocation of AIDS health resources and balance urban development and AIDS prevention. In addition, AIDS awareness, detection, and intervention among Guangxi youths need to be strengthened.

## Introduction

According to the Joint United Nations Programme on HIV/AIDS (UNAIDS), there are an average of 4000 new HIV infections worldwide every day, approximately 27.5% of which occur in young people aged 15-24 years [1]. In China, students accounted for 19% of cases among youths aged 15‐24 years; 82% of the students were infected through homosexual contact [2]. In the past few years, the number of college students newly diagnosed with HIV in China has increased by 30% to 50% annually [3]. Young people were active in sexual behaviors and had more unprotected sexual behaviors, which would increase the risk of HIV transmission [4,5]. HIV/AIDS shortens the lives of young people and imposes an enormous economic burden on individuals and their families [6]. Young people living with HIV often face social discrimination and stigma [7], leading to problems such as low mood and low self-esteem. Therefore, it is necessary to carry out research on HIV/AIDS and prevention among the Chinese youth population.

Due to differences in economic development, population density, and meteorological factors, there are spatial differences in the distribution of infectious diseases [8]. It has been shown that the transmission and distribution of HIV/AIDS exhibit heterogeneity in space and time [9]. Compared with classical linear regression model analysis, the Bayesian spatiotemporal model can correct for the relationship between influencing factors and disease through spatiotemporal effects and then analyze disease time, space, and related elements to improve the accuracy of the model [10]. Population movements in densely populated areas promote the spread of HIV and make these areas vulnerable to the epidemic [11]. The decline in gross domestic product (GDP) has harmed social indicators such as life expectancy, access to education, future income, and a decline in health services, leading to the further spread of HIV infection [12]. The spatiotemporal analysis makes it possible to visually show the changing trends and spatiotemporal distribution of HIV incidence.

This study used a Bayesian spatiotemporal model to analyze the spatiotemporal distribution pattern of HIV among youths aged 15‐24 years in Guangxi from 2014 to 2021. Additionally, we explored the effects of population, GDP per capita, population density, number of health technicians, and road mileage per unit area.

## Methods

### Ethical Considerations

This study was approved by Guangxi Zhuang Autonomous Region Center for Disease Control and Prevention Institutional Review Board (GXIRB2016-0047-3). The data used in this study were from the National Case Reporting Database (NCRD) of the Chinese Comprehensive Response Information Management System of HIV/AIDS (CRIMS), which is a national information system for the management of infectious diseases. The extraction and analysis of these data were reviewed and approved by the Ethics Review Committee and were in accordance with ethical requirements. The procedures followed were in accordance with the ethical standards of the responsible committee (Guangxi Zhuang Autonomous Region Center for Disease Control and Prevention).

### Study Area

As one of the special economic provinces, Guangxi (full name: Guangxi Zhuang Autonomous Region, [104°26‐112°04′ E, 20° 54′-26°24′N]), located in southwestern coastal areas in China and bordering Vietnam, plays an essential role in southwestern coastal areas. It has 14 administrative cities and 111 counties or urban districts, with a land area of 237,600 km^2^ and a resident population of 50.37 million as of 2021. We used the district (county) level as the geographical unit, which is appropriate to study spatial clustering and Bayesian spatiotemporal analysis in Guangxi (Multimedia Appendix 1).

### Data Source

The data for the reported cases of HIV/AIDS among young people aged 15‐24 years in Guangxi from January 2014 to December 2021 were from the Chinese Comprehensive Response Information Management System of HIV/AIDS. The demographic, socioeconomic, and health institution variables analyzed were obtained from the Guangxi Statistical Yearbook. A vector map of Guangxi at the county level was obtained from the National Geomatics Center of China.

We changed the units of some of the data to improve the final presentation as follows: (1) the permanent population at year-end (10,000 persons), (2) GDP per capita (1000 yuan=US $140.40), (3) population density (100 persons per km^2^), (4) number of health technicians (100 persons), and (5) road mileage per unit area (km).

### Statistical Analysis

#### Data Preprocessing

We assessed the number of youths at the district (county) level from 2014 to 2021 through the proportion of the population aged between 15 and 24 in Guangxi, based on the statistical data from the Seventh National Census in 2020.

According to the descriptive statistics of the variables analyzed preliminarily, there are 26 missing values (2.93%) in GDP per capita, 34 missing values (3.83%) in road mileage per unit area, and 11 missing values (1.24%) in the number of health technicians. The missing values of variables were imputed in the statistical software R (version 4.2.1; R Foundation for Statistical Computing). The imputation was performed by considering the trends of each variable over time within different regions [13]. The functions impute_lm and impute_rf from the simputation package were applied when the variable varies linearly with the year and nonlinearly, respectively [14].

#### Incidence Estimates

The ArcGIS 10.8 (ESRI Inc) software was used to describe the spatial distribution of reported HIV/AIDS incidence among youths in Guangxi.

#### Spatial Autocorrelation Analysis

##### Overview

Spatial autocorrelation can analyze the correlation of epidemics in different spatial locations and measure the degree of aggregation of attribute values of spatial units [15]. GeoDa (version 1.10.0.8; Center for Spatial Data Science) was used to conduct a spatial autocorrelation analysis of reported HIV/AIDS among youths in Guangxi. In GeoDa 1.10.0.8 software, the 2014‐2021 Guangxi youth AIDS data were matched with 111 districts (counties) to establish a database.

##### Global Spatial Autocorrelation

Moran I suggests whether there is a cluster and the size of the cluster in the spatial distribution of HIV/AIDS among youths in Guangxi. The global Moran I usually indicates a high level of spatial autocorrelation in the whole region and takes values in the range [−1,1]. For Moran I>0, there is a positive spatial correlation between regions; for example, high values are adjacent to high values, and low values are adjacent to low values. For Moran I<0, there is a negative spatial correlation between regions, such as high values adjacent to low values. For Moran I=0, there is good independence among regions and no spatial correlation. Z values were obtained by hypothesis testing, and differences were considered statistically significant when *P*<.05.

##### Local Spatial Autocorrelation

Local indicators of spatial association (LISA) indicate the spatial correlation of the components in the study area [16]. There are 4 types of local spatial clustering in LISA clustering maps: “High-High,” “Low-Low,” “Low-High,” and “High-Low.” The first 2 types indicate that the observed values in a region are similar to the surrounding region. The remaining 2 types indicate spatial outliers, indicating that a high value is surrounded by adjacent low values or that a low value is surrounded by high neighboring values [17].

### Bayesian Spatiotemporal Analysis

We used the Bayesian spatiotemporal model proposed by Rushworth et al [18] to analyze the impact of social and economic factors on the reported incidence of HIV/AIDS among those aged 15 to 24 years in Guangxi from 2014 to 2021. The model is an extension of the Bayesian spatiotemporal generalized linear mixed model with a spatiotemporal autoregressive process to allow for local spatial heterogeneity (adaptive smoothing) rather than global smoothing.

We assumed that the number of reported HIV/AIDS cases in the *d-*th district in the *t-*th year followed a Poisson distribution:


(1) $$y_{dt}∼Poisson(\lambda_{dt}),\lambda_{dt}=e_{dt}\theta_{dt}\;\;for\;d=1,...,k,\;t=1,...,n$$

Let $y_{dt}$ denote the reported number of HIV/AIDS cases and $e_{dt}$ denote the expected number of HIV/AIDS cases in year *t* in district *d*. $\theta_{dt}$ denotes the ratio of the number of actual cases to the number of expected cases in year *t* in district *d*, which is the mean log relative risk. The Bayesian spatiotemporal model is modeled as follows:


(2) $$\ln\theta_{dt}=\beta_{0}+\sum_{k=1}^{4}x_{ik}\beta_{k}+\Psi_{dt},$$

where $\beta_{0}$ is the intercept,* x*_i_(*i*=1,2,3,4) represents influencing variables (GDP per capita, Population density, Road mileage per unit area, and number of health technicians), *β_k_* denotes the regression coefficients of corresponding variables, and *ψ_dt_* is the random effect for local authority *d* and time period *t*.

The model parameters were estimated using Markov chain Monte Carlo simulation with Gibbs sampling through the function ST.CARar from the CARBayesST package in R (version 4.2.1; R Foundation for Statistical Computing) [19,20]. Values of the posterior mean and 95% CI were then stored and summarized for analysis [21]. For a more detailed description of the model, refer to the supplementary material accompanying this paper (Multimedia Appendix 2).

### Sensitivity Analysis

Given that over 50% of the influencing variables data were missing from Xiufeng district, we applied a random forest algorithm for imputation to address this issue.

## Results

### Spatiotemporal Statistical Analysis

A total of 4638 HIV/AIDS cases among youths were reported in Guangxi from 2014 to 2021. During 2014‐2021, the average incidence of HIV/AIDS in Nanning was 19.94/100,000, ranking first. The average incidence in Hechi was the lowest (5.95/100,000). The average incidence in Nanning was 3.35 times that of Hechi in the past 8 years. Nanning had the highest number of cases (n=1387), and the second and third were Guilin (n=435) and Yulin (n=419), respectively. Fangchenggang had the lowest cumulative number of cases (n=86). The number of youths with HIV/AIDS in Nanning is 16.13 times higher than that in Fangchenggang (Table 1).

**Table 1.** The number of reported HIV/AIDS cases among youths and incidence by city, Guangxi, 2014‐2021.

City	Cases per city, n/N^a^^b^ (per 100,000)								Total
	2014	2015	2016	2017	2018	2019	2020	2021	
Nanning	113/860,024(13.14)	130/860,453(15.11)	149/796,828(18.70)	199/807,111(24.66)	165/818,480(20.16)	216/828,714(26.06)	177/987,545(17.92)	238/996,605(23.88)	1387/6,955,760(19.94)
Liuzhou	48/458,394(10.47)	69/457,672(15.08)	57/446,660(12.76)	53/451,320(11.74)	49/456,025(10.75)	39/460,121(8.48)	49/469,734(10.43)	34/471,099(7.22)	398/3,671,055(10.84)
Guilin	57/592,606(9.62)	55/600,504(9.16)	52/565,165(9.20)	57/570,623(9.99)	41/573,797(7.15)	72/576,821(12.48)	59/557,087(10.59)	42/557,933(7.53)	435/4,594,593(9.47)
Wuzhou	17/352,041(4.83)	25/347,844(7.19)	25/340,566(7.34)	21/342,706(6.13)	28/345,384(8.11)	19/347,178(5.47)	18/318,688(5.65)	16/318,937(5.02)	169/27,133,044(6.23)
Beihai	9/180,945(4.97)	15/187,072(8.02)	23/185,459(12.40)	8/187,669(4.26)	17/189,554(8.97)	20/191,890(10.42)	15/209,367(7.16)	9/211,263(4.26)	116/15,432,168(7.52)
Fangchenggang	16/106,286(15.05)	14/105,575(13.26)	11/104,819(10.49)	12/106,083(11.31)	14/107,561(13.02)	7/108,723(6.44)	6/118,178(5.08)	6/119,239(5.03)	86/87,646,750(9.81)
Qinzhou	60/439,168(13.66)	58/446,231(13.00)	42/365,908(11.48)	54/370,082(14.59)	42/372,835(11.27)	37/375,058(9.87)	20/373,061(5.36)	36/373,558(9.64)	349/3,115,957(11.20)
Guigang	40/567,614(7.05)	56/532,321(10.52)	25/488,780(5.11)	36/493,676(7.29)	46/497,490(9.25)	18/499,927(3.60)	16/487,617(3.28)	27/490,844(5.50)	264/4,058,213(6.51)
Yulin	37/645,388(5.73)	62/708,922(8.75)	55/649,449(8.47)	70/655,631(10.68)	58/660,022(8.79)	48/663,192(7.24)	40/654,877(6.11)	49/656,197(7.47)	419/5,293,675(7.92)
Baise	35/443,885(7.88)	46/450,959(10.20)	52/408,467(12.73)	39/411,435(9.48)	47/414,018(11.35)	47/416,049(11.30)	22/403,480(5.45)	29/403,029(7.20)	317/33,513,025(9.46)
Hezhou	25/272,304(9.18)	16/274,166(5.84)	19/230,027(8.26)	18/232,057(7.76)	22/233,851(9.41)	19/235,284(8.08)	11/226,833(4.85)	9/228,661(3.94)	139/19,331,278(7.19)
Hechi	18/437,194(4.12)	22/440,646(4.99)	35/394,792(8.87)	32/397,557(8.05)	29/400,061(7.25)	22/402,081(5.47)	15/386,138(3.88)	20/385,777(5.18)	193/3,244,254(5.95)
Laibin	31/244,130(12.70)	30/267,373(11.22)	26/248,282(10.47)	17/250,325(6.79)	29/252,051(11.51)	31/253,224(12.24)	15/234,370(6.40)	18/234,438(7.68)	197/1,984,190(9.93)
Chongzuo	27/235,239(11.48)	25/236,255(10.58)	17/233,468(7.28)	26/235,453(11.04)	18/236,875(7.60)	29/238,105(12.18)	11/235,961(4.66)	16/235,555(6.79)	169/18,869,350(8.96)

a n: The number of reported HIV/AIDS cases.

b N: The number of population aged 15‐24 years.

**Figure 1.**
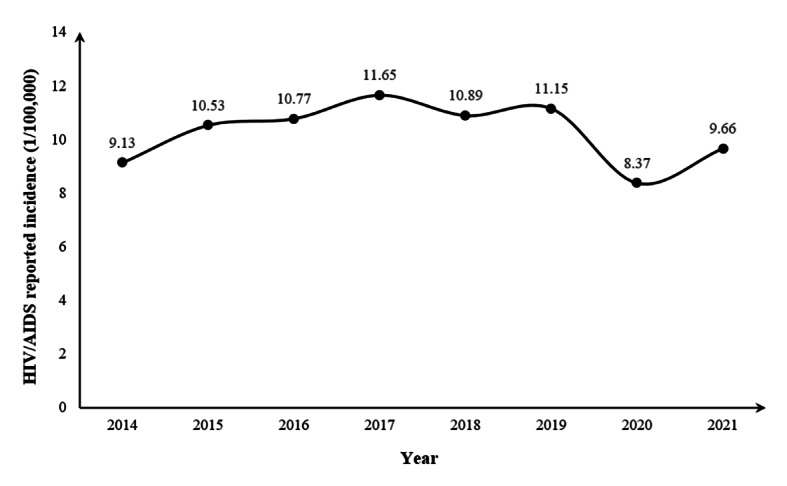
Reported incidence of HIV/AIDS among youths in Guangxi, 2014‐2021.

The reported incidence of HIV/AIDS cases among youths in Guangxi increased from 9.13/100,000 in 2014 to 11.15/100,000 in 2019 and then plummeted to a low of 8.37/100,000 in 2020, followed by a small increase to 9.66/100,000 in 2021 (Figure 1). The number of cases in each city showed an increasing trend from 2014 to 2015 and then decreased. The maximum number was reported in 2017 with 642 cases, among which Nanning ranked first with 199 cases, accounting for 24.66% (Table 1).

**Figure 2.**
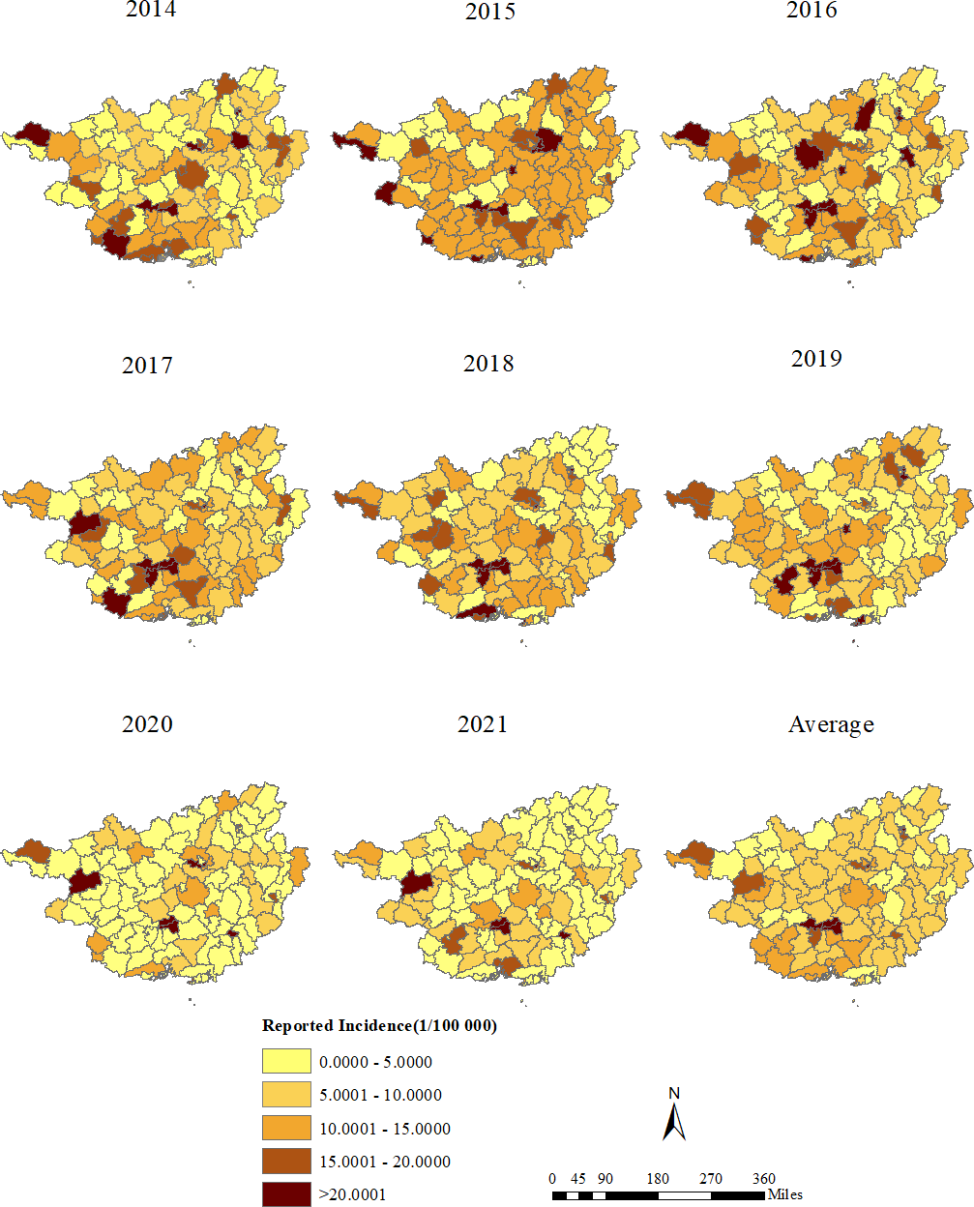
Reported incidence (rate per 100,000 population) of HIV/AIDS among youths at the district (county) level, Guangxi, 2014‐2021. Darker areas mean there is a higher reported incidence of HIV/AIDS among youths.

Figure 2 shows the changes in HIV infection among young people in Guangxi from 2014 to 2021, with darker colors representing a greater reported incidence. In 2014‐2021, youth HIV/AIDS cases were reported in all districts and counties of Guangxi, but they were unevenly distributed. Areas with reported incidences higher than 20 (1/100,000) were mainly concentrated in northwestern, southwestern, and some central areas. Of these, the districts (counties) with an average reported incidence higher than 2 (1/100,000) were Xixiangtang, Xingning, Qingxiu, Chengzhong, and Diecai. In contrast, districts (counties) with low average reported incidence rates (<5, 1/100,000) were mainly concentrated in Hechi, Laibin, Guigang, and Guilin. After 2020, the reported incidence of HIV/AIDS among youths in most districts (counties) of Guangxi was reduced to less than 5 (1/100,000).

### Bayesian Analysis

A Bayesian spatiotemporal model was used to detect factors associated with HIV/AIDS incidence among youths in Guangxi. Table 2 shows the posterior means with 95% CIs for the variables associated with youth HIV/AIDS. The reported incidence of HIV/AIDS among youths was negatively significantly associated with road mileage per unit area (km) at a posterior mean of −0.510 (95% CI −0.818 to 0.209). The reported incidence of HIV/AIDS among youths was positively associated with population density (100 persons) at a posterior mean of 0.025 (95% CI 0.012‐0.038), with the number of health technicians (100 persons) having a posterior mean of 0.007 (95% CI 0.004‐0.009). These results suggested that population density (100 persons) and the number of health technicians (100 persons) are positively related to the reported incidence of HIV/AIDS among youths. Road mileage per unit area (km) was negatively related to the reported incidence of HIV/AIDS among youths (Table 2).

Sensitivity analyses indicated that the estimates of the posterior distribution of the Bayesian spatiotemporal model were robust. Table 3 shows the results of the Bayesian spatiotemporal modeling when deleting the Xiufeng district due to missing values exceeding 50%. It suggested that population density and the number of health technicians are positively related to the reported incidence of HIV/AIDS among youths. In contrast, when the XiuFeng district was retained, road mileage per unit area was negatively associated with the reported incidence of HIV/AIDS among youths.

**Table 2.** Bayesian estimation of socioeconomic factors for reported incidence of HIV/AIDS among youths, Guangxi, 2014‐2021.

Variates	RRa^a^ (95% CI)	Posterior mean (95% CI)	RRb^b^ (95% CI)
GDP^c^ per capita (1000 yuan=US $140.40)	1.150 (1.017 to 1.328)	0.001 (−0.002 to 0.005)	1.051 (0.944 to 1.170)
Population density (100 persons)	1.200 (1.154 to 1.342)	0.025 (0.012 to 0.038)	1.218 (1.097 to 1.348)
Road mileage per unit area (km)	0.932 (0.904 to 0.954)	−0.510 (−0.818 to 0.209)	0.881 (0.817 to 0.948)
number of health technicians (100 persons)	1.150 (1.106 to 1.238)	0.007 (0.004 to 0.009)	1.204 (1.126 to 1.285)

a RRa: estimated relative risk of the univariate Bayesian spatiotemporal model.

b RRb: estimated relative risk of the multivariate Bayesian spatiotemporal model.

c GDP: gross domestic product.

**Table 3.** Bayesian estimation of socioeconomic factors for reported HIV incidence among youths in Guangxi, excluding Xiufeng District, 2014‐2021.

Variates	RRa^a^ (95% CI)	Posterior mean (95% CI)	RRb^b^ (95% CI)
GDP per capita (1000 yuan=US $140.40)	1.172 (1.132 to 1.250)	0.002 (−0.001 to 0.005)	1.067 (0.957 to 1.183)
Population density (100 persons)	1.172 (1.154 to 1.196)	0.020 (0.006 to 0.033)	1.152 (1.042 to 1.268)
Road mileage per unit area (km)	0.998 (0.960 to 0.1000)	−0.268 (−0.573 to 0.020)	0.937 (0.871 to 1.007)
number of health technicians (100 persons)	1.194 (1.181 to 1.252)	0.007 (0.004 to 0.009)	1.204 (1.126 to 1.285)

a RRa: estimated relative risk of the univariate Bayesian spatiotemporal model.

b RRb: estimated relative risk of the multivariate Bayesian spatiotemporal model.

### Spatial Autocorrelation Analysis

Global spatial autocorrelation analysis of the mean annual reported incidence of HIV/AIDS among youths in Guangxi showed that except for 2020 (Moran I=0.0234, *P*=.13) and 2021 (Moran I=0.0691, *P*=.06), Moran I from 2014 to 2019 was in the range of 0.114‐0.297. At the district (county) level, the reported incidence of HIV among youths in Guangxi exhibited significant spatial and regional clustering from 2014 to 2021 (Table 4).

**Figure 3.**
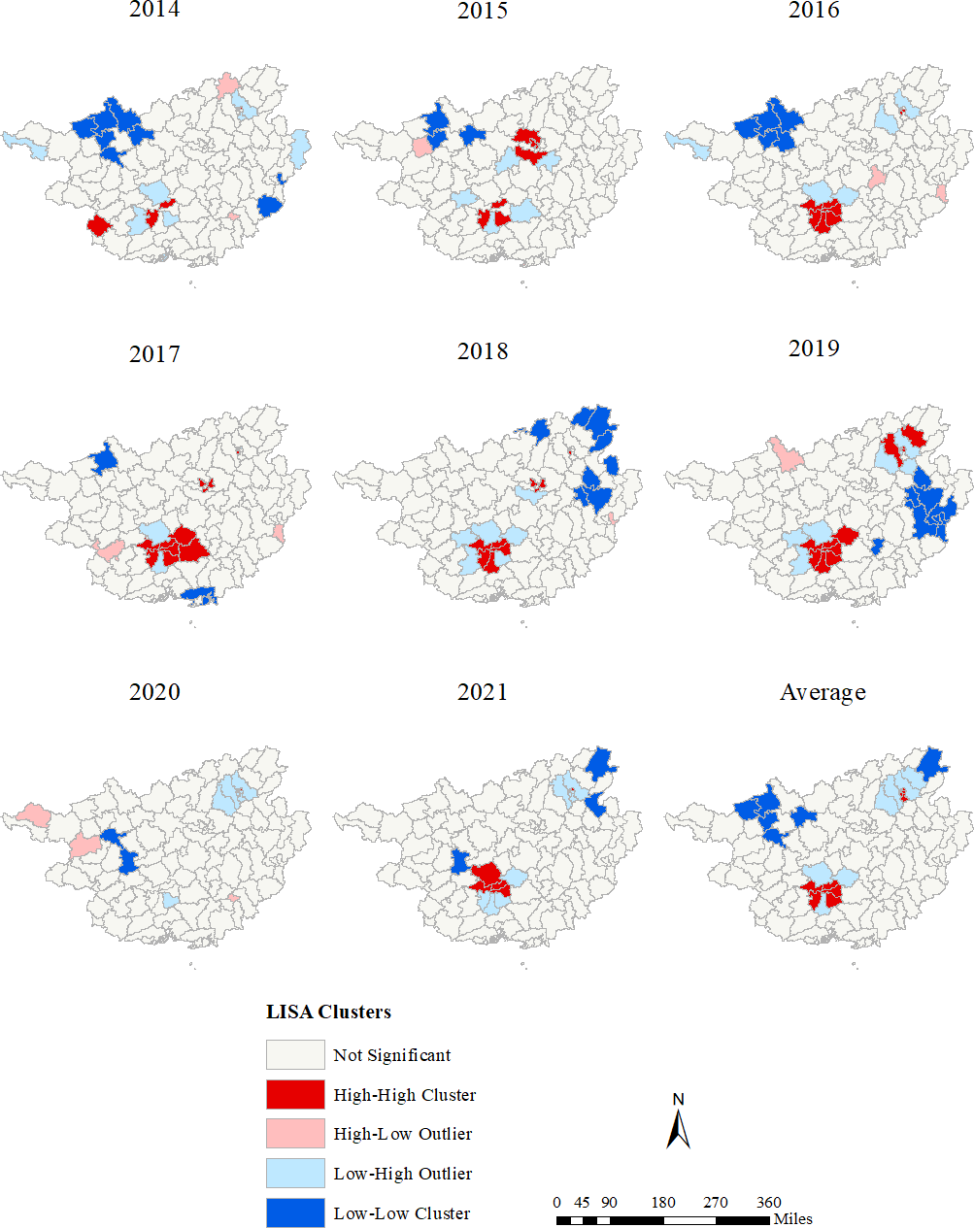
Yearly local indicators of spatial association (LISA) cluster maps for reported incidence of HIV/AIDS among youths, Guangxi, 2014‐2021. Four different colors represent 4 types of local spatial clustering: High-High cluster, Low-Low cluster, Low-High outlier, and High-Low outlier.

LISA analysis can effectively identify the hot spots (High-High) and cold spots (Low-Low) of HIV/AIDS (Figure 3). The distribution of HIV/AIDS among youths in Guangxi is spatially aggregated, with hot spots concentrated in the southwest and cold spots moving from northwest to northeast. Specifically, the hot spots mainly include Xixiangtang, Xingning, Qingxiu, Jiangnan, Yongning, Diecai, Qixing, Xiangshan, and Yanshan; the cold spots mainly include Leye, Tian’e, Fengshan, Bama, Jinchengjiang, and Quanzhou.

**Table 4.** The global spatial autocorrelation of HIV/AIDS among youths in Guangxi, China, 2014‐2021.

Year	Moran I	E (I)	Mean (SD)		Z value	*P* value
2014	0.1136	−0.0091	−0.0075 (0.0598)		2.0234	.03
2015	0.1271	−0.0091	−0.0114 (0.0600)		2.3085	.02
2016	0.1613	−0.0091	−0.011 (0.0600)		2.8701	.003
2017	0.2439	−0.0091	−0.0062 (0.0598)		4.1861	.001
2018	0.2438	−0.0091	−0.0108 (0.0589)		4.3231	.002
2019	0.2974	−0.0091	−0.012 (0.0556)		5.5608	.001
2020	0.0234	−0.0091	−0.009 (0.0382)		0.8464	.13
2021	0.0691	−0.0091	−0.0122 (0.0459)		1.7688	.06
Average	0.2748	−0.0091	−0.0126 (0.0540)		5.3169	.002

## Discussion

### Principal Findings

From 2014 to 2021, a total of 4638 cases of HIV/AIDS infection among youths were reported in Guangxi. The reported incidence of HIV/AIDS among youths in Guangxi increased from 9.13/100,000 in 2014 to 11.15/100,000 in 2019 and then decreased to a low of 8.37/100,000 in 2020, followed by a small increase to 9.66/100,000 in 2021. In 2016‐2019, the population density in Guangxi districts (counties) grew faster, and the reported incidence of HIV/AIDS among youths in Guangxi peaked in 2017 (11.65/100,000) and continued at a higher level. The emergence of this phenomenon was related to Guangxi’s HIV/AIDS prevention and control policy. In 2015, Guangxi launched a 5-year second round of HIV/AIDS prevention and treatment projects [22]. The project has strengthened HIV/AIDS awareness and increased HIV testing among higher education students. Some long-standing but unaware HIV infections and HIV/AIDS cases can be detected earlier [23], resulting in a significant increase in reported incidence. In 2020‐2021, the reported incidence of HIV/AIDS among youths in Guangxi was significantly reduced, and most of the spatially aggregated districts (counties) disappeared. During the epidemic of COVID-19 in Guangxi, the reported incidence of HIV/AIDS among youths decreased by 24.90% compared with 2019. However, in the previous 6 years, the reported incidence of HIV/AIDS among youths in Guangxi continued to increase. This may be related to epidemic prevention and control measures adopted by the Guangxi Zhuang Autonomous Region government, such as closed management and restrictions on the movement of people [24,25]. First, these measures may reduce the timeliness and accessibility of testing for HIV-infected youths, thereby affecting HIV testing among youths. Second, possible changes in the behavioral characteristics of people are associated with HIV infection, such as sexual behavior patterns and population movements [26]. Because of reduced population mobility, youths had less contact with each other, thus reducing the chance of HIV transmission.

The districts (counties) with high HIV/AIDS prevalence among youths were Xixiangtang, Xingning, Qingxiu, Chengzhong, and Diecai. These areas are the gathering place of Guangxi’s colleges and universities, with a higher concentration of young students. Therefore, it is recommended that these regions strengthen the allocation of medical and health resources for HIV/AIDS prevention and control, balance urban development with HIV/AIDS prevention, and strengthen HIV/AIDS publicity, detection, and intervention among youths.

The Bayesian spatiotemporal model combines the embedded time information, spatial information, parameter uncertainty (prior distribution), and related spatiotemporal factors, solves the estimation bias caused by spatial structure, and makes the estimation more stable and reliable [27]. In the present study, the Bayesian spatiotemporal model was used to analyze the spatiotemporal distribution and influencing factors of the reported incidence of HIV/AIDS among youths in Guangxi by combining it with demographic data from 2014 to 2021. The reported incidence of HIV/AIDS among youths was negatively significantly related to road mileage per unit area (km) at a posterior mean of −0.510 (95% CI −0.818 to 0.209). Typically, road miles per unit area are relative to socioeconomic status. Youth groups living in socioeconomically prosperous areas are likely to have better health awareness and easier access to HIV/AIDS health services and prevention measures. The reported incidence of HIV/AIDS among youths was positively related to population density (100 persons) at a posterior mean of 0.025 (95% CI 0.012‐0.038), with the number of health technicians (100 persons) having a posterior mean of 0.007 (95% CI 0.004‐0.009). Studies based on a Bayesian spatiotemporal model showed that population density was positively correlated with the incidence of hand-foot-mouth disease, cholera, and other infectious diseases [28,29]. Previous studies have shown that high social activities and opportunities to make friends in high-density groups will increase the frequency of contact with infectious sources [30], thus promoting the spread of HIV/AIDS. In Guangxi, the population density increased from 2014 to 2021, and the risk of AIDS among youths increased. The number of health technicians can reflect medical resources [31], which are very important for the detection of HIV-infected persons. The more abundant the medical resources are, the higher the timeliness and accessibility of HIV testing. Timely HIV testing with high accessibility helps to identify more young people with HIV/AIDS.

### Limitations

First, since the youth population data were calculated based on the Seventh National Census in 2020, there might be small discrepancies with the actual data. Second, the delay or lag between the reported number of HIV/AIDS infections and the exact number of HIV/AIDS infections inevitably leads to the difference in relative risk [23]. Finally, as an ecological study, ecological fallacies are inevitable due to factors such as individual differences within a group. In the future, more accurate data on the youth population could be collected, and other influencing factors could be considered for further study. However, this study provided a scientific basis for the formulation of prevention and treatment strategies for HIV/AIDS among Chinese youths. It will help the health sector to better allocate HIV/AIDS medical resources and better carry out HIV prevention and control among the youth population.

### Conclusions

In Guangxi, current HIV/AIDS prevention and control among young people should focus on areas with a high risk of disease. It is suggested to strengthen the allocation of HIV/AIDS health resources and balance urban development and HIV/AIDS prevention. In addition, HIV/AIDS awareness, detection, and intervention among Guangxi youths need to be strengthened.

## Supplementary material

10.2196/53361 Multimedia Appendix 1 Guangxi in China geographical position and Guangxi city, district (county) list.

10.2196/53361 Multimedia Appendix 2 A detailed description of the Bayesian spatiotemporal model.

## Abbreviations

GDP: gross domestic product
LISA: local indicators of spatial association
UNAIDS: Joint United Nations Programme on HIV/AIDS
