# Supplementary material for: The Distribution and Associated Factors of HIV/AIDS Among Youths in Guangxi, China, From 2014 to 2021: Bayesian Spatiotemporal Analysis
Source: JMIR Public Health Surveill. 2024 Sep 27;10:e53361. doi: 10.2196/53361 (PMC11452016; doi:10.2196/53361)
Supplement: Multimedia Appendix 2 [file publichealth-v10-e53361-s002.docx]

# The Distribution and Associated Factors of HIV/AIDS among Youths in Guangxi, China, From 2014 to 2021: Bayesian Spatiotemporal Analysis

**Na Wang^1,2,†^, Juntong Li^1,†^, Runxi Zhang^1,†^, Qiuying Zhu^2^, Huanhuan Chen^2^, Jinghua Huang^2^, Dongni Ding^2^, Chunying Li^2^, Yuhua Ruan^3^, Mei Lin^2,*^, Shengkui Tan^1,*^, Guanghua Lan^2,*^**

^1^Guangxi Key Laboratory of Environmental Exposomics and Entire Lifecycle Health, School of Public Health, Guilin Medical University, Guilin, China

^2^Guangxi Key Laboratory of Major Infectious Disease Prevention Control and Biosafety Emergency Response, Guangxi Center for Disease Control and Prevention, Nanning, China

^3^State Key Laboratory of Infectious Disease Prevention and Control (SKLID), Chinese Center for Disease Control and Prevention (China CDC), Collaborative Innovation Center for Diagnosis and Treatment of Infectious Diseases, Beijing, China

^†^These authors contributed equally

^*^Corresponding authors:

Dr. Mei Lin, 18 Jinzhou Road, Nanning, China. E-mail: [gxlinmei@126.com](mailto:gxlinmei@126.com)

Dr. Shengkui Tan, 1 Zhiyuan Road, Guilin, China Email: [861177191@163.com](mailto:wangna2413@163.com)

Dr. Guanghua Lan, 18 Jinzhou Road, Nanning, China Email: [lgh605@163.com](mailto:wangna2413@163.com)

### Bayesian Spatiotemporal Analysis

We used the Bayesian spatiotemporal model proposed by Rushworth et al to analyze the impact of social and economic factors on the reported incidence of HIV/AIDS among those aged 15 to 24 in Guangxi from 2014 to 2021. We assumed that the number of reported HIV/AIDS cases in the *d-*th district in the *t-*th year followed a Poisson distribution:

$y_{dt}$~Poisson($\lambda_{dt}$), $\lambda_{dt}$=$e_{dt}\theta_{dt}$ for *d*=1,…,k, *t*=1,…,n (1)

Let $y_{dt}$ denote the reported number of HIV/AIDS cases and $e_{dt}$ denote the expected number of HIV/AIDS cases in year *t* in district *d*. $\theta_{dt}$denotes the ratio of the number of actual cases to the number of expected cases in year *t* in district *d*, which is the mean log relative risk. The Bayesian spatiotemporal model is modeled as follows:

ln$\theta_{dt}$=$\beta_{0}$+ $\sum_{k=1}^{4} x_{ik}\beta_{k}$ +$\Psi_{dt}$, (2)

where
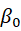
 is the intercept, $x_{i}$( *i*=1,2,3,4) represents influencing variables (GDP per capita, Population density, Road mileage per unit area, and number of health technicians), $\beta_{k}$ denotes the regression coefficients of corresponding variables, and $\Psi_{dt}$ is the random effect for local authority *d* and time period *t*.

The model is that proposed by Rushworth et al. (2014)[1], and represents the spatio-temporal structure with a multivariate first order autoregressive process with a spatially correlated precision matrix. This model is appropriate if one wishes to estimate the evolution of the spatial response surface over time without forcing it to be the same for each time period. The model specification is given below

$\Psi_{dt}$=$\Phi_{dt}$, (3)

$\Phi_{t}$|$\Phi_{t-1}$~ N($\rho_{T}\Phi_{t-1}$,$\tau^{2}$**Q**(**W**, $\rho_{S})^{-1}$) t=2,…,8,

$\Phi_{1}$~ N(**0**,$\tau^{2}$**Q**(**W**, $\rho_{S})^{-1}$) ,

$\tau^{2}$ ~ Inverse-Gamma(a, b),

$\rho_{S}$, $\rho_{T}$ ~ Uniform(0, 1).

In this model $\Psi_{t}$ = ($\Phi_{1t}$, . . . , $\Phi_{kt}$) is the vector of random effects for time period t, which evolve over time via a multivariate first order autoregressive process with temporal autoregressive parameter ρ_T_ . The temporal autocorrelation is thus induced via the mean ρ_T_$\Phi_{t-1}$, while spatial autocorrelation is induced by the variance τ^2^**Q**(**W**, ρ_S_)^−1^. The corresponding precision matrix **Q**(**W**, ρ_S_) was proposed by Leroux et al. (2000)[2] and corresponds to the CAR models used in the other models above. The algebraic form of this matrix is given by

**Q**(**W**,
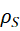
) =$\rho_{S}$(diag[**W1**]-**W**)+(1-$\rho_{S}$)**I**, (4)

where **1** is the k × 1 vector of ones and **I** is the k × k identity matrix. In common with all other models, the random effects are zero-mean centered, while flat and conjugate priors are specified for ($\rho_{S}$, $\rho_{T}$) and
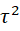
, respectively, with (a = 1, b = 0.01) being the default values for the latter.

We utilized the *ST.CARar* function from the *CARBayesST* package in R (version 4.2.1; R Foundation for Statistical Computing) to estimate the model parameters through Markov chain Monte Carlo (MCMC) simulations incorporating Gibbs sampling. The MCMC procedure involved running 2200,000 samples, with the initial 200,000 samples discarded during the burn-in period to ensure stabilization. To minimize autocorrelation within the Markov chain, the remaining samples were thinned by a factor of 1000, yielding 2,000 samples suitable for inference.

Table S1. Fitness information of the multivariate Bayesian spatiotemporal model across 111 districts (counties) in Guangxi, China, from 2014 to 2021.

|  | Posterior Mean | 2.5% | 97.5% | n.effective | Geweke.diag |
| --- | --- | --- | --- | --- | --- |
| (Intercept) | -0.3991 | -0.6067 | -0.2036 | 2000.0 | -0.7 |
| Gdpp | 0.0014 | -0.0016 | 0.0045 | 2130.2 | 0.2 |
| Dp | 0.0252 | 0.0118 | 0.0376 | 2135.4 | -0.9 |
| Kms | -0.5101 | -0.8177 | -0.2094 | 2000.0 | 0.5 |
| Htech | 0.0065 | 0.0037 | 0.0091 | 2000.0 | -0.1 |
| tau2 | 0.3966 | 0.2728 | 0.5378 | 2000.0 | 1.5 |
| rho.S | 0.3622 | 0.1772 | 0.5743 | 2000.0 | 0.4 |
| rho.T | 0.7328 | 0.6243 | 0.8416 | 2000.0 | -1.0 |

DIC = 3773.007 p.d = 343.2959 LMPL = -1941.175

Gdpp: GDP per capita (1000yuan)

Pd: population density (100 persons)

Kms: road mileage per unit area(km)

NHtech: the number of health technicians (100 persons)

tau2 (τ²): the parameter represents the spatial precision

rho.S: the spatial autocorrelation coefficient

rho.T: the temporal autocorrelation coefficient

DIC: Deviance Information Criterion

LMPL: Log Marginal Predictive Likelihood

Table S2. Fitness information of the multivariate Bayesian spatiotemporal model across 110 districts (excluding XiuFeng District) in Guangxi, China, from 2014 to 2021.

|  | Posterior Mean | 2.5% | 97.5% | n.effective | Geweke.diag |
| --- | --- | --- | --- | --- | --- |
| (Intercept) | -0.5424 | -0.7451 | -0.3301 | 2000.0 | 0.2 |
| Gdpp | 0.0018 | -0.0012 | 0.0048 | 2000.0 | -0.2 |
| Dp | 0.0195 | 0.0057 | 0.0331 | 1946.5 | 0.0 |
| Kms | -0.2678 | -0.5734 | 0.0200 | 1840.0 | -0.5 |
| NHtech | 0.0068 | 0.0044 | 0.0091 | 1748.8 | 0.0 |
| tau2 | 0.4328 | 0.3171 | 0.5676 | 2000.0 | 0.7 |
| rho.S | 0.5831 | 0.3573 | 0.7922 | 2000.0 | 0.7 |
| rho.T | 0.7055 | 0.5918 | 0.8184 | 1517.8 | 1.0 |

DIC = 3715.192 p.d = 319.5159 LMPL = -1907.776

Table S3. Bayesian estimation of socioeconomic factors for reported HIV incidence among youths in Guangxi, excluding Xiufeng District, 2014-2021.

| Variates | RRa(95%CI) | Posterior Mean(95%CI) | RRb(95%CI) |
| --- | --- | --- | --- |
| GDP per capita (1000yuan) | 1.172(1.132-1.250) | 0.002(-0.001-0.005) | 1.067(0.957-1.183) |
| Population density (100 persons) | 1.172(1.154-1.196) | 0.020(0.006-0.033) | 1.152(1.042-1.268) |
| Road mileage per unit area (km) | 0.998(0.960-0.1000) | -0.268(-0.573-0.020) | 0.937(0.871-1.007) |
| number of health technicians (100 persons) | 1.194(1.181-1.252) | 0.007(0.004-0.009) | 1.204(1.126-1.285) |

Table S4. The cluster of HIV/AIDS reported cases detected by using the space-time scan statistic.

| Cluster type | Time frame | Radius (km) | Cluster areas | Observed | Expected | ODE | LLR | RR | P value |
| --- | --- | --- | --- | --- | --- | --- | --- | --- | --- |
| Most likely | 2018-2021 | 19.17 | QingXiu, XingNing | 443 | 69.94 | 6.33 | 460.38 | 6.90 | <0.001 |
| Secondary | 2020-2021 | 0 | XiuFeng | 71 | 3.74 | 18.98 | 142.21 | 19.26 | <0.001 |
| 2nd Secondary | 2020-2021 | 0 | YuZhou | 52 | 20.85 | 2.49 | 16.47 | 2.51 | <0.001 |
| 3rd Secondary | 2015 | 36.81 | LuZhai, YuFeng | 26 | 8.98 | 2.90 | 10.66 | 2.91 | 0.022 |

DE: observed/expected, LLR: log likelihood ratio, RR: relative risk

Using Kulldorff’s spatiotemporal scan statistics, we identified several significant clusters of HIV/AIDS reported cases over different time frames and geographic areas. The most likely cluster was identified in QingXiu and XingNing from 2018 to 2021, with a radius of 19.17 km.

### Reference

1. Rushworth A, Lee D, Mitchell R. A spatio-temporal model for estimating the long-term effects of air pollution on respiratory hospital admissions in Greater London. Spatial and spatio-temporal epidemiology. 2014 Jul;10:29-38.[doi:10.1016/j.sste.2014.05.001] Epub 2014/08/13.

2. Leroux BG, Lei X, Breslow N, editors. Estimation of Disease Rates in Small Areas: A new Mixed Model for Spatial Dependence2000; New York, NY: Springer New York.
